# Clinical, endoscopic, and demographic characteristics of idiopathic duodenal ulcers compared with helicobacter pylori positive ulcers

**DOI:** 10.22088/cjim.14.2.179

**Published:** 2023

**Authors:** Manijeh Ebrahimzadeh, Khadijeh Haghshenas, Mehrdad Kashifard, Javad Shokri-Shirvani

**Affiliations:** 1 Student Research Committee, Babol University of Medical Sciences, Babol, Iran; 2 Department of Internal Medicine, Babol University of Medical Sciences, Babol, Iran; 3 Department of Internal Medicine, Babol University of Medical Sciences, Babol, Iran

**Keywords:** Idiopathic Ulcer, Helicobacter Pylori, Duodenal Ulcer, Endoscopy

## Abstract

**Background:**

*Helicobacter pylori* infection is the most common cause of peptic ulcer disease. However, the prevalence rates of non-helicobacter pylori idiopathic peptic ulcers have increased over the past few years. This study aims to compare the characteristics of *Helicobacter pylori*-positive with idiopathic duodenal ulcers.

**Methods:**

A cross-sectional cohort study was conducted on 950 patients which were excluded from the analysis process duo to the concomitant presence of gastric ulcer, malignancy, Zollinger Ellison syndrome, Crohn's disease, esophageal varices, history of taking anti-Helicobacter pylori therapy, and history of taking NSAID or aspirin. Eventually, 647 subjects were enrolled for the analysis process. In this case, these subjects were divided into two groups: (I) *Helicobacter pylori*-positive ulcer group and (II) *Helicobacter pylori*-negative and non-NSAID (idiopathic) ulcer group.

**Results:**

The findings showed that 417 patients (64.5%) had duodenal ulcers induced by *Helicobacter pylori*, and 111 patients (17.1%) had *Helicobacter pylori*-negative and non-NSAID ulcers. The mean ages of patients in *Helicobacter pylori*-positive and idiopathic ulcer groups were 39±15 and 42±17, respectively. In this case, 33 patients (29.7%) with idiopathic ulcers and 56 patients (25.1%) with *Helicobacter pylori*-positive ulcers had upper gastrointestinal bleeding. Also, 22 patients (21%) with idiopathic ulcers and 31 patients (16.5%) with *Helicobacter pylori*-positive ulcers had multiple duodenal ulcers

**Conclusion:**

The present study demonstrated that the idiopathic ulcers included 17.1% of duodenal ulcers. Also, it was concluded that patients with idiopathic ulcers were predominantly male with an age range older than the other group. In addition, patients in this group had more ulcers.

Generally, peptic ulcer disease (PUD) has often been a common cause of hospitalization and mortality (1). This disease substantially occurs due to the imbalance between invasive factors (i.e., gastric acid, bile salts, and Helicobacter pylori (H. pylori) infection) and protective mechanisms (i.e., mucus). H. pylori causes various gastrointestinal disorders, including gastritis, duodenal and gastric ulcer, and gastric adenocarcinoma (2, 3). The literature survey showed that the worldwide prevalence of PUD was about 6 to 15%, and this value was around 12.5% in Iran. Duodenal ulcers are responsible for a significant portion of peptic ulcers (4, 5). H. pylori and non-steroidal anti-inflammatory drugs (NSAIDs) drugs are the primary risk factors in PUD. Besides, if a patient is free of these two menaces, this situation is called idiopathic peptic ulcer disease (IPUD) (6).

Although the use of antibiotics and improved health facilities have declined the prevalence of H. pylori-induced peptic ulcers, IPUD has increased in Asia and worldwide over the last decades (1, 7). The IPUD outbreak was 13.11% in Iran, and duodenal ulcers were the most frequent problem (8). Some studies proved that IPUD caused recurrent bleeding and mortality rates higher than others. Although proton pump inhibitor (PPI) and H2-receptor antagonist (H2RA) drugs have successfully been utilized to reduce the complications of peptic ulcers, these types of ulcers are less responsive to these drugs (9, 10). Also, patients with IPUD are at high risk of recurrent ulcers, which leads to a poor prognosis (11). In addition, idiopathic perforated ulcers significantly have a high rate (12).Dental Xia et al. (13) reported that the prevalence of idiopathic duodenal ulcers in China was 17.4%. In this study, the patients in the idiopathic group were older than NSAID and *H. pylori* groups. Besides, these patients had more comorbidities, which was statistically significant. Also, they proved that IPUD patients were more likely to show no symptoms such as pain or discomfort in the epigastric region. Charpignon et al. (14) demonstrated that age and comorbidities were independent risk factors for IPUD, and its prevalence was 21.4%. Also, a study has been performed in Italy and reported that the prevalence of IPUD was about 4% (15). In this case, NSAIDs and *H. pylori* infections were offered as the leading causes of peptic ulcers. In 2015, Kanno et al. (16) reported that the prevalence of IPUD was 12%. In this case, the IPUD patients were more prone to develop gastrointestinal symptoms, despite taking PPI and H2RA drugs. Also, they indicated that the IPUD patients were older than the *H. pylori*-related ulcers group and younger than the NSAID-related group.

In 2017, a study revealed that subjects with vulnerable personalities had a higher risk of developing idiopathic ulcers than others (17). The present study is focused on investigating and comparing the clinical, endoscopic, and demographic characteristics of idiopathic duodenal ulcers with *H. pylori-*positive ulcers in the Babol geographical region, northern Iran. The primary purpose of this study revolves around a better understanding of idiopathic peptic ulcers for faster diagnosis and reduction of IPUD complications.

## Methods

This cross-sectional and single-center cohort study was performed from December 2005 to March 2012 at Shahid Beheshti Hospital, Babol, Iran. This study included all patients above 16 years old who underwent an upper endoscopy and were diagnosed with duodenal ulcers. A total of 6622 consecutive patients were referred to the endoscopy unit. In this case, about 950 patients suffered from duodenal ulcers. Also, 95 patients were excluded from the study due to the concomitant presence of a gastric ulcer, malignancy, Zollinger Ellison syndrome, Crohn's disease, esophageal varices, and history of taking anti-*Helicobacter pylori* therapy in the preceding 12 months. Among the remaining 855 patients, the researchers had access to 647 H. pylori infection status reports. The collected demographic data were age, sex, smoking habit, and opium consumption (inhaled or taken three times a week for at least six months). Also, the patient's clinical symptoms were recorded through a physician in a standard questionnaire at the admission time. These symptoms were epigastric pain, nausea, vomiting, fullness, and bleeding from the upper gastrointestinal tract (hematemesis or melena). 

**Ethical Approval** **:** Informed consent has been obtained from all patients before participating in the study. Also, this research was approved by the Research Ethics Committee of Babol University of Medical Sciences under ethics number 8929928. 

**Endoscopy and** ***H. pylori*** **status:** Patients underwent endoscopy under sedation. Then, the number and location of ulcers were recorded on a standard report form by a specialized gastroenterologist. Two tissue samples were taken from the antrum and the corpus of the stomach and then stained with H&E or Giemsa. Also, the presence or absence of *H. pylori* infection was reported by the specialized pathologist. Then, patients with positive test results for *H. pylori* were classified as *H. pylori*-positive ulcers. Also, patients who have taken NSAIDs in the past 30 days were classified as NSAID-related ulcers. This classification was performed regardless of the presence or absence of *H. pylori*. Thus, these patients were excluded from the study. In addition, patients with duodenal ulcers, not associated with *H. pylori *or NSAID, were classified as idiopathic ulcers. Finally, the parameters were evaluated based on the two groups of *H. pylori*-positive and idiopathic ulcers.

**Statistical Analysis:** The collected data have been analyzed comparatively between the two groups of idiopathic ulcer and H. pylori-positive. The analysis process was performed using the chi-squared test (with Yates’ correction, if required) or Fisher’s exact test. The Mann–Whitney U-test has been employed to determine the difference between the location and number of ulcers in the groups of patients with duodenal ulcers. SPSS Statistics version 16 has been utilized for the analysis process. All calculated p-values were two-tailed, and the significance level was set to 0.05 (P < 0.05).

## Results

**Figure 1:**
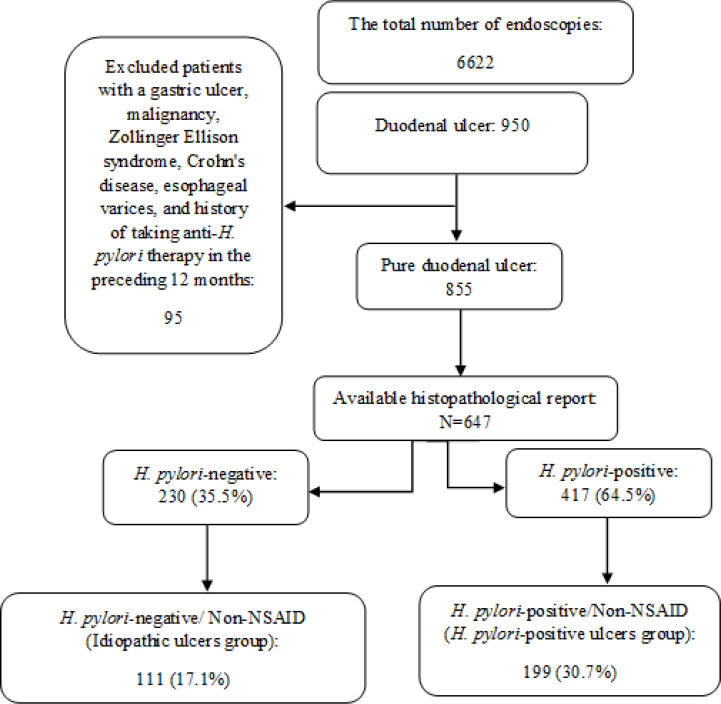
Flowchart for grouping of the studied patients

A total of 6622 patients underwent upper endoscopy within six years. In this regard, 950 patients (14.3%) were diagnosed with duodenal ulcers. Also, 95 patients who did not pass the inclusion criteria were excluded from the study. Among the remaining 855 patients with pure duodenal ulcer, 208 patients had no histopathological report of *H. pylori* infection. Lastly, 647 patients were eligible for the study. In the patients’ cohort, 417 participants (64.5%) were *H. pylori*-positive, and 230 participants (35.5%) were *H. pylori*-negative. Besides, 199 patients (30.7%) in the *H. pylori*-positive group and 111 patients (17.1%) in the *H. pylori*-negative subjects had no history of the use of NSAID or aspirin. Figure 1 depicts a flowchart and summarizes the cohort grouping design in the studied patients.

The mean overall age of patients was 47±17.6. Besides, the mean ages in the *H. pylori*-positive and idiopathic ulcer groups were 39±15 (18-84 years) and 42±17 (16-80 years), respectively. Among the remaining 647 patients, 396 (61.2%) and 251 (38.8%) patients were males and females, respectively. Also, the *H. pylori*-positive and idiopathic groups included 120 (60.3%) and 78 (70.3%) male patients, respectively. Among all patients, 76 (11.7%) participants were smokers. In this regard, 24 (12.1%) and 15 (13.5%) smoker patients were in the *H. pylori*-positive and idiopathic ulcer groups, respectively. Also, 8 (4%) and 5 (4.5%) patients were opium users in the *H. pylori*-positive and idiopathic ulcer groups, respectively. A total of 169 (26.1%) patients were referred to the hospital with upper gastrointestinal bleeding. Among these patients, 56 (28.1%) and 33 (29.7%) patients were in the *H. pylori*-positive and idiopathic ulcer groups, respectively. 

In the *H. pylori*-positive group, 77 (72%) and 30 (28%) patients had at least one ulcer in the anterosuperior and posteroinferior duodenum regions, respectively. In the idiopathic ulcer group, 33 (67.3%) and 16 (32.7%) patients had an ulcer in similar locations. Also, 82 (78.8%) out of 104 patients had one ulcer. It was associated with 152 (83.1%) out of 183 patients in the *H. pylori*-positive group. Also, 22 (21.2%) and 31 (16.9%) patients had multiple ulcers (i.e., two or more) in the idiopathic ulcer and *H. pylori*-positive groups, respectively. Among the 647 patients, 337 (52.1%) patients had a history of NSAID use in the preceding 30 days. In this case, 218 (33.6%) patients were *H. pylori*-positive. Table 1 provides the demographic and endoscopic information in the *H. pylori*-positive and idiopathic groups. 

**Table 1:** The clinical, endoscopic, and demographic data for H .pylori-positive and idiopathic ulcers groups

Features		Groups		p-value
		Idiopathic ulcers Count (%)	H. pylori-positive ulcers Count (%)	
Mean Age (Year)		42±17	39±15	-
Sex	**Male**	78 (70.3%)	120 (60.3%)	0.086
	**Female**	33 (29.7%)	79 (39.7%)	
Smoker		15 (13.5%)	24 (12.1%)	0.75
Opium Consumption		5 (4.5%)	8 (4%)	0.86
Upper Gastrointestinal Bleeding		33 (29.7%)	56 (28.1%)	0.7
Number of Ulcers	**1**	82 (78.8%)	152 (83.1%)	0.83
	**2 or more**	22 (21.2%)	31 (16.9%)	
Location	**Anterosuperior**	33 (67.3%)	77 (72%)	0.077
	**Posteroinferior**	16 (32.7%)	30 (28%)	

## Discussion

Most Among the 647 participants studied in the present study, 17.1% of patients suffered from idiopathic peptic ulcers, and about 64.5% of patients were *H. pylori*-positive. Characteristics such as male gender, older age, and multiple ulcers were associated with idiopathic ulcers, but the difference was not statistically significant. In this regard, Xia et al. (13) performed a study and reported that 66% of patients with duodenal ulcers were *H. pylori*-positive, and 17% had idiopathic ulcers. The results of the present study regarding the prevalence of idiopathic ulcers were consistent with Xia’s study. In 2007, Yakoob et al. (18) reported that 29% of patients with duodenal ulcers were *H. pylori*-negative and had no history of NSAID intake. In 2011, 29.6% of Indian patients with duodenal ulcers were *H. pylori*-negative (19). 

In the present study, the mean ages of patients were 39±15 and 42±17 in the *H. pylori*-positive and idiopathic ulcer groups, respectively. In this regard, Hung et al. (20) reported the mean ages of patients, which were matched with the present study. We found Idiopathic ulcers more common in male patients undergoing upper endoscopy, but it was not significant with p = 0.086. In 2019 Dore et al. (21), male patients had significantly higher rates of idiopathic peptic ulcers (55.8% male vs. 44.2% female).

In the *H. pylori*-positive group, 28.1% of subjects had upper gastrointestinal bleeding. This value was less than the idiopathic ulcer group (29.7%) but the difference was not statistically significant. In 2004, Adamopoulos et al. (22) reported *H. pylori*-negative patients with duodenal ulcers, showing significantly higher rates of upper gastrointestinal bleeding than *H. pylori*-positive patients. In a seven-year cohort performed by Wong in 2009 (9), the bleeding rates were 42.3 and 11.2% in the idiopathic ulcer and *H. pylori*-positive groups, respectively. These results showed a significant difference between these two groups. Similar to other studies, despite a higher rate of smoking in idiopathic ulcer patients (12.1% compared to 13.5%), we did not find smoking as a risk factor for idiopathic peptic ulcers (21, 23). Xia et al. (24) represented that multiple ulcers were more common in the non-helicobacter pylori and non-NSAID group which is consistent with our findings but we did not demonstrate the significant relationship despite Xia et al. In the present study, the ulcers located in the anterosuperior region were more than in the other areas. In this case, the values were 72 and 67.3% in the *H. pylori*-positive and idiopathic ulcer groups, respectively. But no significant difference was observed between these two groups. It is expected that health facility improvement in Iran gradually decreases the prevalence of *H. pylori*. In the present study, the value of 17.1% for idiopathic duodenal ulcers corresponds to this issue.

Ghadimi et al. (25) accomplished a study in 2007 and reported that the prevalence rates of *H. pylori* were 81.1 and 77.5% in women and men, respectively. In the present study, the prevalence of *H. pylori* was 64.5%, which confirmed the above results. It is concluded that various study designs cause different rates of idiopathic ulcers. In other words, these differences lead to statistical variations (29.6% and 4% versus 17.1%) (19, 15). However, these findings indicated the existence of reality called idiopathic ulcers with distinct prognoses. In the present study, histopathological examination of gastric biopsy specimens has been utilized to assess the presence of *H. pylori* infection. Some patients may be false negatives, but the sensitivity and specificity of the histology test is 90-95%.The findings showed that in symptomatic patients, duodenal ulcers included 17.1% of idiopathic ulcers. This study found no significant difference between characteristics of idiopathic ulcers and *H. pylori*-positive ulcers. It is recommended to conduct more extensive and prospective studies with a more precise data collecting system to minimize the missing data. Comprehensible questionnaires can result in more accurate patient history (i.e., PPI or NSAIDs consumption).
